# Beyond antipsychotic efficacy and toward an individualized therapeutic strategy: analysis of the systemic, metabolic, and inflammatory effects of olanzapine, haloperidol, and their combination in schizophrenic patients

**DOI:** 10.3389/fphar.2026.1727959

**Published:** 2026-02-03

**Authors:** Nayef Samah Alharbi, Noha Alaa Hamdy, Esam M. Aboubakr, Mansour Alharbi, Mostafa A. Ali, Ghaleb Alharbi, Ahmed Ibrahim ElMallah

**Affiliations:** 1 Eradah and Mental Health Hospital, Ministry of Health, Buraidah, Saudi Arabia; 2 Department of Clinical Pharmacy and Pharmacy Practice, Faculty of Pharmacy, Alexandria University, Alexandria, Egypt; 3 Department of Pharmacology and Toxicology, Faculty of Pharmacy, South Valley University, Qena, Egypt; 4 Department of Psychiatry, College of Medicine, Qassim University, Buraidah, Saudi Arabia; 5 Department of Psychiatry, College of Medicine, Assuit University, Assiut, Egypt; 6 Department of Clinical Pharmacy, College of Pharmacy, Shaqra University, Shaqra, Saudi Arabia; 7 Department of Pharmacology and Toxicology, Faculty of Pharmacy, Alexandria University, Alexandria, Egypt

**Keywords:** haloperidol, inflammatory, metabolic, olanzapine, schizophrenia

## Abstract

**Introduction:**

Olanzapine (OLZ), haloperidol (HALP), and their combination, are widely used antipsychotics; the majority of studies focuses on their therapeutic efficacy, leaving significant gaps in understanding their systemic impacts. Hence, the present study was conducted to investigate their systemic, metabolic, and inflammatory impact on schizophrenic patients.

**Methods:**

A total of 75 schizophrenic patients and 25 healthy volunteers were involved and monitored over a six-month period. Study groups were as follow; normal control, OLZ (20 mg/day), HALP (10 mg/day), and OLZ (20 mg/day) + HALP (5 mg/day). The parameters of metabolic, inflammatory, and neuronal transmitters, along with cardiovascular, hepatic, and renal functions were determined.

**Results:**

In this study we found that OLZ and HALP produced a noticeable decrease in potassium and chloride ions, while their combination decreased potassium, chloride and calcium. OLZ and OLZ + HALP significantly prolonged QTc, while OLZ and HALP individual administration increased SBP and CK-MP respectively. HbA1C levels not significantly affected by tested drugs, while OLZ produced a significant reduction in LDL and HDL levels, while OLZ + HALP modestly decreased LDL levels. renal assessment revealed a significant increase in both creatinine and urea concentrations in the OLZ + HALP group compared to other groups, whereas hepatic function showed no significant differences between the treated groups. OLZ significantly decreased total bilirubin and increased ALP activity, while HALP significantly reduced total and direct bilirubin levels. OLZ and OLZ + HALP produced a significant increase in body weight and waist circumference, which was not found in HALP-treated patients. Schizophrenic patients had reduced dopamine levels that was not significantly affect by OLZ or OLZ + HALP administration, while HALP administration normalized dopamine levels. Schizophrenic patients had significantly higher levels of serotonin compared to controls, that was normalized by our tested drugs. Ghrelin levels were significantly lower in schizophrenic patients, and it was significantly increased by HALP administration. Leptin hormone significantly elevated in schizophrenics, and was significantly decreased by HALP administration. Schizophrenic patients exhibited markedly elevated levels of IL-17, IL-6, and TNF-α, that dramatically decreased by tested drugs, and the combined regimen showed the highest impact.

**Conclusion:**

This study provides valuable insights into the systemic, metabolic, and inflammatory effects of tested drugs, support the individualized therapeutic approaches in schizophrenic patients to optimize clinical outcomes and minimizing long-term systemic risks.

## 1 Introduction

Schizophrenia affects millions of people worldwide. It is a serious, chronic mental disorder causing significant disruptions in thinking, perception, and emotional responsiveness, leading to severe disability (Tandon et al., 2024). Schizophrenia is one of the top 15 leading causes of disability globally, and it is associated with a dramatic reduction in life expectancy, with an average potential life lost due to physical illnesses and suicide risk (Howes et al., 2024). The burden is widespread but varies by region, with higher prevalence in adults and substantial treatment gaps, especially in low-income regions (Howes et al., 2024; McCutcheon et al., 2023). This highlights schizophrenia as a major global public health challenge requiring greater awareness, early intervention, and access to care worldwide (McCutcheon et al., 2023).

The pharmacological treatment of schizophrenia primarily targets dopamine and, in the case of newer agents, also serotonin receptors to reduce psychotic symptoms like hallucinations and delusions (McCutcheon et al., 2023). First-generation (“typical”) antipsychotics—such as haloperidol and chlorpromazine block dopamine D2 receptors, while second-generation (“atypical”) agents like olanzapine, risperidone, and aripiprazole have broader receptor profiles and generally cause fewer motor side effects (Goff, 2021). The lifetime treatment is usually required to prevent relapse and maintain functional stability (Howes et al., 2024).

Although antipsychotic medications represent the cornerstone in managing schizophrenia, many patients experience severe side effects, including restlessness, endocrine issues, sexual dysfunction, weight gain, sedation, and cardiovascular risks (Leucht et al., 2024). This side effect burden often leads to high rates of medication nonadherence, which can result in relapse, hospitalization, prolonged illness, and increased suicide risk (Pillinger et al., 2023). Approximately 50% of patients exhibit nonadherence, greatly complicating effective treatment (Pillinger et al., 2023). The problem of schizophrenia treatment with antipsychotics centers on balancing their significant benefits in symptom control against the considerable challenges posed by adverse effects, medication adherence, and long-term health risks (Emsley, 2025).

Clinicians face a dilemma in choosing between typical and atypical antipsychotics. Typical (first-generation) drugs are associated with neurological morbidities like extrapyramidal symptoms and tardive dyskinesia, whereas atypical (second-generation) drugs reduce these but carry risks for metabolic syndrome, diabetes, obesity, and cardiovascular disease (McCrone et al., 2021). This creates a delicate risk-benefit calculation, as the long-term health impact of atypicals can be severe. Continuous monitoring and individualized treatment decisions are essential (Yeo et al., 2021). The dilemma also includes the choice about maintenance treatment (Stahl et al., 2021). While maintenance with antipsychotics reduces relapse risk substantially, it also prolongs exposure to adverse effects (Veras, 2022). Some studies support dose reduction or even discontinuation in selected patients, but discontinuation carries a high relapse risk, underscoring the trade-off between efficacy and harm (Veras, 2022).

Olanzapine and haloperidol are widely used antipsychotics for treating schizophrenia but represent two different classes with distinct profiles. Olanzapine is an atypical antipsychotic effective across positive, negative, and cognitive symptoms, generally preferred for its lower risk of extrapyramidal symptoms (EPS) (Meftah et al., 2020). It is commonly dosed between 10 and 20 mg daily and has been associated with improvements in quality of life and social functioning (Meftah et al., 2020). However, olanzapine carries a notable risk of metabolic side effects, including weight gain, diabetes, and dyslipidemia (Lazzari et al., 2017).

Haloperidol, a typical antipsychotic, primarily targets positive symptoms such as hallucinations and delusions and is highly effective but often limited by its higher risk of Extrapyramidal symptoms (EPS), tardive dyskinesia, and other movement disorders (Schrijver et al., 2016). It is available in oral and long-acting injectable forms, useful for adherence challenges (Schrijver et al., 2016).

Comparative studies show similar efficacy of olanzapine and haloperidol in symptom control but differ significantly in side effect profiles (Ibragimov et al., 2024): olanzapine is linked with sedation and metabolic risks, while haloperidol has more neurological adverse effects (Boettger et al., 2015). Thus, olanzapine tends to be favored for long-term maintenance in many patients, while haloperidol remains valuable, especially when rapid symptom control or injectable formulations are needed (Buoli et al., 2016). Treatment choice must balance efficacy with tolerance of side effects and patient-specific factors (Buoli et al., 2016).

The metabolic, cardiac, and immunological impact of OLZ and HALP administration on schizophrenic patients has not fully investigated. While antipsychotic polypharmacy is typically reserved for treatment-resistant cases and is not a first-line recommendation, it remains a prevalent practice in real-world clinical settings. Despite its use, data regarding the specific cumulative metabolic and inflammatory risks of combining olanzapine and haloperidol are nearly absent. Hence, the present study was conducted. Most research focuses on monotherapy or comparisons rather than combinations. Hence, the present study was conducted to evaluate the effect of OLZ, HALP, and OLZ + HALP on patients’ metabolic, cardiac, and inflammatory parameters in addition to their impact on kidney and hepatic functions.

## 2 Materials and methods

### 2.1 Ethical aspects

The study was conducted in accordance with the ethical guidelines established in the last iterations of the Declaration of Helsinki by the World Medical Association in 1991 and 1996. Furthermore, it adhered to the principles of good clinical practice and the relevant laws and regulations of the Ministry of Health in the Kingdom of Saudi Arabia, which offered superior protection for the individuals concerned. The study protocol, informed consent forms, and all pertinent study-related documentation were approved by an independent ethics committee at the Ministry of Health in the Kingdom of Saudi Arabia (National Committee of Bio&Med Ethics (NCBE)) under approval No: H-04-Q-001. Study participants were enrolled after receiving a comprehensive description of the study design and submitting written informed consent.

### 2.2 Study settings and design

The study followed a cohort prospective study design. Patients admitted to the Qassim Mental Health Hospital–Saudi Arabia and diagnosed with schizophrenia, in the period from July 2023 to April 2024, were checked for eligibility. Diagnosis was based on the criteria outlined in the Diagnostic and Statistical Manual of Mental Disorders, Fifth Edition (DSM-5), American Psychiatric Association, 2013. Patients’ demographic data were determined as follow: patients’ age, gender (female/male), marital status (married/unmarried), and occupational state (employed/unemployed).

### 2.3 Inclusion criteria

Patients were required to exhibit at least a moderate level of current positive and/or negative symptoms at the time of examination, operationalized as a minimum BPRS score of 8 on core positive-symptom items and a SANS total score ≥20. This threshold was chosen to ensure inclusion of patients with active schizophrenia requiring antipsychotic treatment and to reduce heterogeneity due to near-remitted or subclinical presentations.

### 2.4 Exclusion criteria

The study exclusion criteria were; diagnosis of a DSM-5 substance-related disorder, history of seizures, history of leukopenia, leukocyte count of less than 3.5 ^3/uL and/or neutrophilic granulocyte count of less than 2.0 ^3/uL at the time of study beginning, the use of diuretics or any medication that could affect the determined metabolic, cardiovascular, hepatic, or renal parameters, diabetes, current jaundice, and active hepatitis were among the exclusion criteria. In addition, patients with medical conditions that could influence inflammatory markers (e.g., autoimmune diseases, chronic inflammatory conditions, immunomodulatory treatments), or patients who had previously experienced OLZ or HALP therapy failure because of side effects or ineffectiveness were not involved in the current study.

### 2.5 Study design

In this study, 166 patients were involved, of whom 109 met our inclusion and exclusion criteria. 36 patients were administered OLZ based on their mental health status and the psychiatrist’s recommendation. Four patients chose to continue their treatment and follow-up at a nearby mental health facility due to distance and transportation challenges. Three patients received other medications that could have influenced our outcome results (NSAIDs). Additionally, the psychiatrist modified the treatment plan for four patients during the study. A total of 38 patients received HALP; 5 patients chose to continue their treatment at a nearby mental health facility, 4 patients experienced comorbidities (sever infection and new onset of diabetes) during the study that required the administration of additional medications, potentially influencing the results, From the eligible pool of participants, 25 patients were randomly selected for the final analysis to achieve the pre-determined balanced sample size required for the study design. 35 patients received OLZ + HALP, and 5 of them chose to continue their treatment at a nearby mental health facility. Three patients experienced comorbidities during the study that required the administration of additional medications (antihypertensives and immunosuppressants), potentially influencing the results, and from the eligible pool of participants, 25 patients were randomly selected for the final analysis to achieve the pre-determined balanced sample size required for the study design.

Eligible patients were divided into the following study groups as follows:

1. Normal control group; normal volunteers were involved
2. Olanzapine (OLZ) treated group; patients received OLZ (20 mg/day), and parameters were assessed before the initiation of OLZ, 3 months later, and 6 months after drug administration (Leucht et al., 2020; Wesner et al., 2023)
3. Haloperidol (HALP) treated group; patients received HALP (10 mg/day), and parameters were determined before initiation of the treatment, 3 months later, and 6 months after drug administration (Potkin et al., 2015; Ifteni et al., 2016).
4. Olanzapine (OLZ) + Haloperidol (HALP) treated group; patients were received OLZ (20 mg/day) + HALP (5 mg/day), and parameters were determined before initiation of the treatment, 3 months later, and 6 months after drug administration (Lähteenvuo and Tiihonen, 2021).

### 2.6 Biochemical parameters determination

Standard laboratory techniques were used in the central laboratory of Qassim Mental Health Hospital–Saudi Arabia, to determine the blood levels of urea, creatinine, ALT, AST, total bilirubin, direct bilirubin, albumin, sodium, potassium, magnesium, chloride, calcium, CK-MB, glycosylated hemoglobin, cholesterol, HDL, and LDL.

### 2.7 Test of cardiac performance

A specialized independent cardiologist determined the patient’s heart rate, electrocardiogram (ECG) QTc, systolic and diastolic blood pressure.

### 2.8 Neurotransmitter determination

The patient’s serum levels of serotonin, leptin, dopamine, and ghrelin were determined using a solid-phase sandwich ELISA technique. Kits from BT LAB, Shanghai Korain Biotech, Shanghai, China, were used; the catalog numbers were E1128Hu, E1559Hu, EA0041Hu, and E3091Hu, respectively.

### 2.9 Analysis of the inflammatory mediators by western blot

Laemmli sample buffer with protease inhibitors (ThermoScientific, USA) was used to recover denatured proteins from serum samples, and the total protein concentration of cell lysate was measured using Sigma Aldrich Coomassie protein assay kit #27813.

The concentrations of IL-6, IL-1β, and TNF-α in serum samples were determined using the Western blot technique using Human IL-6 Recombinant Protein (MW: 20.95 kDa) with Catalog # RIL6I (dilution 1:1000), Human IL-1 beta Monoclonal Antibody (dilution 1:300), eBioscience, Thermosientific, USA, and Human TNF-α Recombinant Protein (MW: 17.5 kDa), Catalog #PHC3011 (dilution 1:500), (Gibco, Thermosientific, USA). The membrane was examined at 340 nm using the Analytik Jena, USA UVP Transilluminator gel documentation instrument. For every sample, the protein ratio that is, the ratio of protein concentration to total protein was calculated using Analytica Jena software.

### 2.10 Analysis of statistics

Statistical analyses were conducted using SPSS version 22.0 (IBM Corp, Armonk, NY, USA) and GraphPad Prism (GraphPad Software, San Diego, CA, USA). Data are expressed as mean ± SEM for normally distributed variables or mean ± SD for neurotransmitter and inflammatory parameters. Within-subject longitudinal analysis: To assess changes over time (baseline → 3 months → 6 months) within each treatment group, we employed Friedman’s repeated-measures test (the nonparametric equivalent of repeated-measures ANOVA). When the Friedman test indicated significant within-group changes (p < 0.05), pairwise comparisons between timepoints were performed using Wilcoxon signed-rank test with Bonferroni correction for multiple comparisons. This approach accounts for within-subject correlation across repeated measurements.

Between-group cross-sectional comparisons: To compare the three treatment groups at each individual timepoint (baseline, 3 months, or 6 months separately), we used Kruskal–Wallis test for non-normally distributed data or one-way ANOVA for normally distributed data. When the omnibus test was significant, pairwise group comparisons were performed using Mann–Whitney U test (with Bonferroni correction) or Tukey’s *post hoc* test, respectively.

Between treatment groups at baseline only (schizophrenia patients): Baseline characteristics among the three treatment groups were compared using Kruskal–Wallis or one-way ANOVA as appropriate, to verify group comparability prior to treatment initiation. Statistical significance was defined as p < 0.05 (two-tailed). All tests were performed with appropriate corrections for multiple comparisons where indicated.

Given the large number of biochemical parameters assessed (>70 variables), we adopted a hierarchical testing framework to control Type I error. Primary endpoints were defined *a priori* as:

1. Change in body weight and waist circumference (metabolic safety)
2. Change in QTc interval (cardiovascular safety)
3. Change in inflammatory cytokines (IL-6, IL-17, TNF-α)

For these three co-primary outcome domains, Bonferroni correction was applied (adjusted significance threshold: α = 0.05/3 = 0.017). All other biochemical parameters (electrolytes, renal function, hepatic enzymes, lipid profile, neurotransmitters, and appetite hormones) were designated as exploratory secondary endpoints and analyzed without adjustment for multiple comparisons, with the understanding that findings are hypothesis-generating and require independent validation. Unadjusted p-values are reported for exploratory outcomes with appropriate cautious interpretation.

## 3 Results

### 3.1 The demographic properties

The demographic characteristics of the study participants were as follows: in the normal group, 52% were male and 48% female, 76% were married and 24% unmarried, 72% were employed and 28% unemployed, with a mean age of 44.1 years. In the OLZ-treated group, males and females constituted 52% and 48% respectively; 68% were married, whereas 32% were unmarried; 76% were employed and 24% were unemployed; the mean age was 42.6 years. In HALP- treated group, 56% were male and 44% were female; 72% were married, whereas 28% were unmarried; 68% were employed and 32% were unemployed; the mean age was 46.3 years. In OLZ + HALP combination group, 56% were male and 44% were female; 68% were married, whereas 32% were unmarried; 72% were employed and 28% were unemployed; the mean age was 45.3 years, as shown in Table 1.

**TABLE 1:** participants’ demographic properties.

Demographic properties	Control		OLZ		HALP		OLZ + HALP	
​	N	%	N	%	N	%	​	​
Gender								
Male	13	52	13	52	14	56	14	56
Female	12	48	12	48	11	44	11	44
Marital status								
Married	19	76	17	68	18	72	17	68
Unmarried	6	24	8	32	7	28	8	32
Employment								
Employed	18	72	19	76	17	68	18	72
Unemployed	7	28	6	24	8	32	7	28
​	Mean	SD	Mean	SD	Mean	SD	Mean	SD
Age	44.1	8.7	42.6	9.5	46.3	11.1	45.3	10.5

### 3.2 Effect on patients’ neurotransmitters and appetite-regulating hormones

**FIGURE 1:**
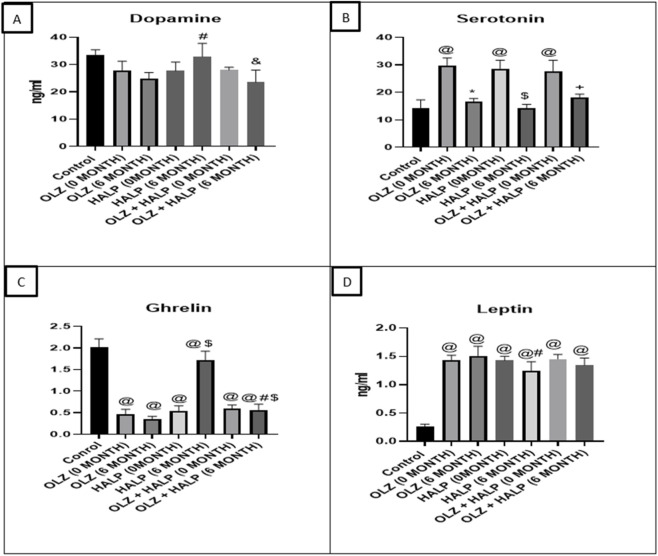
Analysis of biochemical parameters in controls, schizophrenic patients, and schizophrenia patients after treatment: **(A)** dopamine, **(B)** serotonin, **(C)** ghrelin, and **(D)** leptin. ^@^ significantly different in comparison to the control group, * significantly different compared to OLZ (0 months), ^#^ significant different compared to OLZ (6 months), ^$^ significant different compared to HALP (0 months), and significant different compared to HALP (6 months), + significant different compared to OLZ + HALP (0 months). Results are presented as the mean ± SEM (n = 25).

In the present study, schizophrenic patients exhibited markedly reduced dopamine levels compared to the control group. After 6 months of treatment, no significant alterations in dopamine levels were observed among patients receiving OLZ or the OLZ + HALP combination. In contrast patients treated with HALP alone demonstrated normalization of dopamine levels, with significant higher values than those recorded in the OLZ-treated group (Figure 1A).

With respect to serotonin, schizophrenic patients showed significantly elevated serum levels compared to controls. However, treatment with OLZ, HALP, or OLZ + HALP for 6 months resulted in a significant reduction of serotonin levels, nearly restoring them to normal values (Figure 1B).

Concerning ghrelin, patients with schizophrenia exhibited significantly lower serum ghrelin concentrations relative to healthy individuals. Administration of OLZ further decreased ghrelin levels, whereas HALP treatment significantly increased them. Patients receiving the OLZ + HALP combination displayed ghrelin levels significantly lower than those observed in the HALP-only group (Figure 1C).

Regarding leptin, schizophrenic patients presented with markedly elevated serum leptin concentrations—approximately sixfold higher than in normal controls. Treatment with the tested drugs produced no significant effect on leptin levels, except for HALP, which significantly reduced leptin concentrations after 6 months of administration (Figure 1D).

### 3.3 Effect on patients’ inflammatory mediators

**FIGURE 2:**
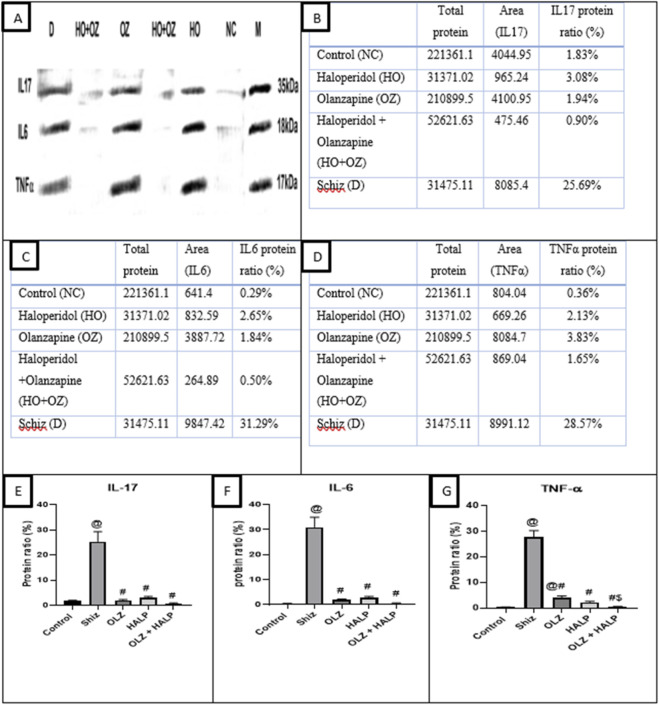
Determination of the inflammatory mediator concentrations in controls, schizophrenic patients, and schizophrenic patients following drugs administration. **(A)** A whole Western blot image, **(B)** Quantifications of Western Blots-stained IL-17 protein, **(C)** Quantifications of Western Blots-stained IL-6 protein, **(D)** Quantifications of Western Blots-stained TNF-α protein, **(E)** representative graph for IL-17 serum level, **(F)** representative graph for IL-6 serum level, **(G)** representative graph for TNF-α serum level. NC = normal control, schiz = D = patients with schizophrenia before treatment; HALP = HO = patients with schizophrenia after 6 months of haloperidol treatment, OLZ = OZ = patients with schizophrenia after 6 months of olanzapine treatment; OLZ + HALP = HO + OZ = patients with schizophrenia after 6 months of olanzapine treatment and haloperidol. ^@^ significantly different in comparison to the control group, ^#^ significant different compared to OLZ (6 months), ^$^ significant different compared to HALP (0 months Results are presented as the mean ± SEM (n = 12).

As illustrated in Figure 2A, patients with schizophrenia exhibited markedly elevated serum concentrations of the pro-inflammatory cytokine IL-17 compared with healthy controls. Treatment with OLZ, HALP, or their combination (OLZ + HALP) for 6 months resulted in near normalization of IL-17 levels, with the combination therapy producing the most pronounced downregulatory effect.

Regarding IL-6, the present study demonstrated that schizophrenic patients had significantly higher serum IL-6 concentrations compared to the control group. Oral administration of OLZ, HALP, or OLZ + HALP for 6 months effectively reduced IL-6 levels, restoring them to near-normal values (Figure 2B).

Regarding the effect on TNF-α, schizophrenic patients showed significantly elevated concentrations of this inflammatory mediator relative to healthy individuals. Six months of treatment with OLZ, HALP, or OLZ + HALP led to a significant reduction in TNF-α levels. Notably, the OLZ + HALP combination exhibited the strongest suppressive effect, whereas OLZ alone exerted the least impact (Figure 2C).

### 3.4 Effect on blood minerals

As presented in Tables 2–6, oral administration of OLZ, HALP, and OLZ + HALP for 6 months resulted in a modest overall decline in blood mineral concentrations, with the effect being most pronounced in the HALP-treated group. Specifically, potassium levels decreased from 4.11 mmol/L to 3.93 mmol/L, while chloride concentrations showed a significant reduction from 103.2 mEq/L to 94.7 mEq/L. In the OLZ-treated group, a significant decrease was observed in both sodium and chloride levels following 6 months of treatment. Moreover, combined administration of OLZ + HALP produced a significant reduction in sodium, calcium, and chloride concentrations.

**TABLE 2:** Comparing the patients’ blood mineral levels before starting treatment with olanzapine, haloperidol, and olanzapine + haloperidol.

Laboratory data (0 months)	Olanzapine group (I) (N = 25)	Haloperidol group (II) (N = 25)	Olanzapine + haloperidol group (III) (N = 25)	
	Mean ± SD	Mean ± SD	Mean ± SD	
Sodium level (mmol/L)	136.3 ± 2.52	137.9 ± 3.17	137.1 ± 3.11	
Potassium level (mmol/L)	4.018 ± 0.28	4.11 ± 0.35	4.01 ± 0.30	
Calcium level (mmol/L)	2.151 ± 0.04	2.27 ± 0.03	2.35 ± 0.07	
Chloride level (mmol/L)	101.4 ± 4.55	103.2 ± 6.28	103.9 ± 6.28	
Magnesium level (mmol/L)	0.808 ± 0.04	0.8 ± 0.06	0.8 ± 0.03	
P value				
​	All groups	I vs. II	I vs. III	II vs. III
Sodium level (mmol/L)	0.896	0.696	0.696	>0.99
Potassium level (mmol/L)	0.988	0.897	0.897	>0.99
Calcium level (mmol/L)	0.946	0.787	0.787	>0.99
Chloride level (mmol/L)	0.789	0.57	0.57	>0.99
Magnesium level (mmol/L)	0.734	0.523	0.523	>0.99

Analyzed by Mann-Whitney U test and Kruskal Wallis Test.

*: Significant difference at P value <0.05.

**TABLE 3:** Comparing the patients’ blood mineral levels following treatment with olanzapine, haloperidol, and olanzapine + haloperidol for 6 months.

Laboratory data ((post 6 months))	Olanzapine group (I) (N = 25)	Haloperidol group (II) (N = 25)	Olanzapine + haloperidol group (III) (N = 25)	
	Mean ± SD	Mean ± SD	Mean ± SD	
Sodium level (mmol/L)	126.2 ± 3.58	132.2 ± 6.18	121 ± 5.67	
Potassium level (mmol/L)	4.042 ± 0.31	3.93 ± 0.42	4.21 ± 0.32	
Calcium level (mmol/L)	2.338 ± 0.12	2.38 ± 0.45	2.13 ± 0.08	
Chloride level (mmol/L)	94.06 ± 3.07	94.7 ± 21.1	92.6 ± 5.35	
Magnesium level (mmol/L)	0.760 ± 0.19	0.76 ± 0.13	0.6 ± 0.05	
P value				
​	All groups	I vs. II	I vs. III	II vs. III
Sodium level (mmol/L)	0.591	0.315	0.778	0.515
Potassium level (mmol/L)	0.594	0.37	0.811	0.419
Calcium level (mmol/L)	0.125	0.656	0.065	0.094
Chloride level (mmol/L)	0.838	0.599	0.623	0.896
Magnesium level (mmol/L)	0.719	0.77	0.599	0.413

Analyzed by Mann-Whitney U test and Kruskal Wallis Test.

*: Significant difference at P value <0.05.

**TABLE 4:** Comparing the patients’ blood mineral levels before starting treatment and after 6 months of olanzapine treatment.

Laboratory data	Basal (0 months) (N = 25)	After 6 months (N = 25)	p value
	Mean ± SD	Mean ± SD	​
Sodium level (mmol/L)	137.9 ± 2.52	126.2 ± 3.58	<0.001*
Potassium level (mmol/L)	4.018 ± 0.28	4.042 ± 0.31	0.723
Calcium level (mmol/L)	2.351 ± 0.04	2.338 ± 0.12	0.502
Chloride level (mmol/L)	104.2 ± 4.55	94.06 ± 3.07	<0.001*
Magnesium level (mmol/L)	0.808 ± 0.04	0.760 ± 0.19	0.433

Analyzed by Wilcoxon test.

*: Significant difference at P value <0.05.

**TABLE 5:** Comparing the patients’ blood mineral levels before starting treatment and after 6 months of haloperidol treatment.

Laboratory data	Basal (0 months) (N = 25)	After 6 months (N = 25)	p value
	Mean ± SD	Mean ± SD	​
Sodium level (mmol/L)	137.9 ± 3.17	132.2 ± 6.18	0.207
Potassium level (mmol/L)	4.11 ± 0.35	3.94 ± 0.42	0.937
Calcium level (mmol/L)	2.27 ± 0.03	2.38 ± 0.45	0.953
Chloride level (mmol/L)	103.2 ± 6.28	94.7 ± 21.1	0.049*
Magnesium level (mmol/L)	0.8 ± 0.06	0.76 ± 0.13	0.115

Analyzed by Wilcoxon test.

*: Significant difference at P value <0.05.

**TABLE 6:** Comparing the patients’ blood mineral levels before starting treatment and after 6 months of olanzapine + haloperidol treatment.

Laboratory data	Basal (0 months) (N = 25)	After 6 months (N = 25)	p value
	Mean ± SD	Mean ± SD	​
Sodium level (mmol/L)	137.1 ± 3.11	121 ± 5.67	0.006*
Potassium level (mmol/L)	4.01 ± 0.30	4.21 ± 0.32	0.624
Calcium level (mmol/L)	2.35 ± 0.07	2.13 ± 0.08	0.021*
Chloride level (mmol/L)	103.9 ± 6.28	92.6 ± 5.35	0.033*
Magnesium level (mmol/L)	0.8 ± 0.03	0.6 ± 0.05	0.674

Analyzed by Wilcoxon test.

*: Significant difference at P value <0.05.

### 3.5 Effect on cardiovascular parameters

With respect to the cardiovascular effects of the tested drugs, baseline measurements revealed that the HALP group exhibited a significantly lower ECG (QTc) interval compared to the OLZ and OLZ + HALP groups. Additionally, diastolic blood pressure was significantly lower in the OLZ + HALP group relative to both the OLZ and HALP groups. After 3 months of follow-up, no significant differences were observed among the treated groups in any of the evaluated cardiovascular parameters. However, upon individual assessment of drug effects after 6 months, OLZ administration resulted in a moderate but significant increase in both ECG (QTc) and systolic blood pressure (SBP). Treatment with HALP led to a moderate elevation in CK-MP levels (from 10.78 ± 3.58 to 14.02 ± 7.86), and a modest increase in QTc interval (mean increase +11.9 m; p = 0.05), although this increase was less pronounced than that observed in the OLZ group. Whereas the OLZ + HALP combination produced a moderately significant increase in ECG (QTc) values (Tables 7-12).

**TABLE 7:** Cardiovascular parameters before starting treatment.

Cardiac parameter (0 months)	Olanzapine group (I) (N = 25)	Haloperidol group (II) (N = 25)	Olanzapine + haloperidol group (III) (N = 25)	
	Mean ± SD	Mean ± SD	Mean ± SD	
ECG (QTc)	404.1 ± 14.5	375.8 ± 30.7	395.4 ± 21	
SBP	119.1 ± 6.74	118.6 ± 9.96	117 ± 9.63	
DBP	76.21 ± 5.48	77.35 ± 5.7	71.9 ± 6.77	
HR	75.14 ± 7.67	75.58 ± 15.7	79.9 ± 8.79	
CKMP	10.03 ± 4.88	9.98 ± 3.58	10.78 ± 3.58	
P value				
​	All groups	I vs. II	I vs. III	II vs. III
ECG (QTc)	0.002*	0.001*	0.06	0.017*
SBP	0.574	0.684	0.284	0.559
DBP	0.025*	0.753	0.028*	0.014*
HR	0.333	0.551	0.116	0.541
CKMP	0.241	0.172	0.172	>0.99

Analyzed by Mann-Whitney U test and Kruskal Wallis Test.

*: Significant difference at P value <0.05.

**TABLE 8:** Effect of olanzapine, haloperidol, and olanzapine + haloperidol on cardiovascular parameters after 3 months of treatment.

Cardiac parameter (post 3 months)	Olanzapine group (I) (N = 25)	Haloperidol group (II) (N = 25)	Olanzapine + haloperidol group (III) (N = 25)	
	Mean ± SD	Mean ± SD	Mean ± SD	
ECG (QTc)	411.5 ± 14	384 ± 26.7	396.5 ± 23.4	
SBP	116.8 ± 4.82	122.2 ± 7.3	115.7 ± 7.72	
DBP	75.1 ± 6.67	73.68 ± 5.91	74 ± 6.98	
HR	76.78 ± 7.89	74.05 ± 16.2	77.57 ± 10.6	
P value				
​	All groups	I vs. II	I vs. III	II vs. III
ECG (QTc)	0.002*	0.001*	0.043*	0.218
SBP	0.017*	0.013*	0.67	0.015*
DBP	0.822	0.853	0.582	0.615
HR	0.616	0.212	0.651	0.749

Analyzed by Mann-Whitney U test and Kruskal Wallis Test.

*: Significant difference at P value <0.05.

**TABLE 9:** Effect of olanzapine, haloperidol, and olanzapine + haloperidol on cardiovascular parameters after 6 months of treatment.

Cardiac parameter (post 6 months)	Olanzapine group (I) (N = 25)	Haloperidol group (II) (N = 25)	Olanzapine + haloperidol group (III) (N = 25)	
	Mean ± SD	Mean ± SD	Mean ± SD	
ECG (QTc)	417 ± 15.1	387.7 ± 31.7	408.3 ± 24.8	
SBP	128.9 ± 9.41	120.1 ± 8.87	116.8 ± 8.95	
DBP	77.35 ± 7.08	75.4 ± 5.88	75 ± 9.86	
HR	79.57 ± 13.2	77.47 ± 7.1	78.65 ± 12.3	
CKMP	14.74 ± 3.47	14.02 ± 1.86	13.5 ± 2.87	
P value				
​	All groups	I vs. II	I vs. III	II vs. III
ECG (QTc)	0.026*	0.011*	0.137	0.161
SBP	0.059	0.334	0.014*	0.215
DBP	0.459	0.23	0.344	0.876
HR	0.674	0.778	0.724	0.311
CKMP	0.232	0.244	0.121	0.502

Analyzed by Mann-Whitney U test and Kruskal Wallis Test.

*: Significant difference at P value <0.05.

**TABLE 10:** Effect of Olanzapine on cardiovascular parameters after 3 and 6 months of treatment.

Cardiac parameter	Basal (0 months) (N = 25)	After 3 months (N = 25)	After 6 months (N = 25)	p value
	Mean ± SD	Mean ± SD	Mean ± SD	​
ECG (QTc)	404.1 ± 14.5	411.5 ± 14	417 ± 15.1	<0.001*
SBP	119.1 ± 6.74	116.8 ± 4.82	128.9 ± 9.41	0.035*
DBP	76.21 ± 5.48	75.1 ± 6.67	77.35 ± 7.08	0.750
HR	75.14 ± 7.67	76.78 ± 7.89	79.57 ± 13.2	0.640
CKMP	10.03 ± 4.88	-	14.74 ± 3.47	0.125

Analyzed by Friedman test and Wilcoxon test.

*: Significant difference at P value <0.05.

**TABLE 11:** Effect of haloperidol on cardiovascular parameters after 3 and 6 months of treatment.

Cardiac parameter	Basal (0 months) (N = 25)	After 3 months (N = 25)	After 6 months (N = 25)	p value
	Mean ± SD	Mean ± SD	Mean ± SD	​
ECG (QTc)	375.8 ± 30.7	384 ± 26.7	387.7 ± 31.7	0.05
SBP	118.6 ± 9.96	122.2 ± 7.3	120.1 ± 8.87	0.199
DBP	77.35 ± 5.7	73.68 ± 5.91	75.4 ± 5.88	0.765
HR	75.58 ± 15.7	74.05 ± 16.2	77.47 ± 7.1	0.590
CKMP	9.98 ± 3.58	-	14.02 ± 1.86	0.047 *

Analyzed by Friedman test and Wilcoxon test.

*: Significant difference at P value <0.05.

**TABLE 12:** Effect of olanzapine + haloperidol on cardiovascular parameters after 3 and 6 months of treatment.

Cardiac parameter	Basal (0 months) (N = 25)	After 3 months (N = 25)	After 6 months (N = 25)<	p value
	Mean ± SD	Mean ± SD	Mean ± SD	​
ECG (QTc)	395.4 ± 21	396.5 ± 23.4	408.3 ± 24.8	0.033*
SBP	117 ± 9.63	115.7 ± 7.72	116.8 ± 8.95	0.779
DBP	71.9 ± 6.77	74 ± 6.98	75 ± 9.86	0.313
HR	79.9 ± 8.79	77.57 ± 10.6	78.65 ± 12.3	0.694
CKMP	10.78 ± 3.58	-	13.5 ± 2.87	0.715

Analyzed by Friedman test and Wilcoxon test.

*: Significant difference at P value <0.05.

### 3.6 Effect of olanzapine, haloperidol, and olanzapine + haloperidol on glycosylated hemoglobin and patients’ lipid profile

As presented in Tables 13–17, no significant differences were observed among the treatment groups in baseline glycosylated hemoglobin or blood lipid concentrations prior to drug administration. Similarly, after 6 months of treatment, there remained no significant intergroup differences in these parameters. However, longitudinal analysis revealed a significant reduction in both LDL and HDL levels within the OLZ-treated group over the study period, whereas the HALP-treated group showed no significant changes in any of the assessed parameters. In contrast, patients receiving the OLZ + HALP combination exhibited a slight decrease in LDL levels following treatment.

**TABLE 13:** Glycosylated hemoglobin and patients’ lipid concentrations before Olanzapine, Haloperidol, and Olanzapine + Haloperidol administration.

Glucose and lipid profile Basal (0 months)	Olanzapine group (I) (N = 25)	Haloperidol group (II) (N = 25)	Olanzapine + haloperidol group (III) (N = 25)	
	Mean ± SD	Mean ± SD	Mean ± SD	
HbA1C (mmol/L)	5.88 ± 0.89	5.86 ± 1.2	5.90 ± 1.22	
Cholesterol (mmol/L)	4.33 ± 0.67	4.2 ± 0.91	4.41 ± 0.95	
LDL (mg/dL)	85.85 ± 11.9	92.05 ± 22.9	90.65 ± 17.9	
HDL (mmol/L)	1.29 ± 0.24	1.28 ± 0.4	1.48 ± 0.32	
P value				
​	All groups	I vs. II	I vs. III	II vs. III
HbA1C (mmol/L)	0.823	0.601	0.601	>0.99
Cholesterol (mmol/L)	0.735	0.512	0.512	>0.99
LDL (mg/dL)	0.918	0.73	0.73	>0.99
HDL (mmol/L)	0.709	0.489	0.489	>0.99

Analyzed by Mann-Whitney U test and Kruskal Wallis Test.

*: Significant difference at P value <0.05.

**TABLE 14:** Effect of olanzapine, haloperidol, and olanzapine + haloperidol on glycosylated hemoglobin and patients’ lipid profile after 6 months of treatment.

Glucose and lipid profile (post 6 months)	Olanzapine group (I) (N = 25)	Haloperidol group (II) (N = 25)	Olanzapine + haloperidol group (III) (N = 25)	
	Mean ± SD	Mean ± SD	Mean ± SD	
HbA1C (mmol/L)	5.59 ± 0.98	6.2 ± 2.1	5.86 ± 1.21	
Cholesterol (mmol/L)	4.43 ± 0.92	3.76 ± 0.85	4.4 ± 0.95	
LDL (mg/dL)	79.15 ± 13.5	79.4 ± 18.4	92.05 ± 22.9	
HDL (mmol/L)	1.09 ± 0.15	1.51 ± 1.74	1.28 ± 0.42	
P value				
​	All groups	I vs. II	I vs. III	II vs. III
HbA1C (mmol/L)	0.999	0.897	0.944	0.922
Cholesterol (mmol/L)	0.718	0.451	0.694	0.616
LDL (mg/dL)	0.321	0.291	0.152	0.64
HDL (mmol/L)	0.491	0.219	0.342	0.572

Analyzed by Mann-Whitney U test and Kruskal Wallis Test.

*: Significant difference at P value <0.05.

**TABLE 15:** Effect of Olanzapine on glycosylated hemoglobin and patients’ lipid profile after 6 months of treatment.

Blood glucose and lipid profile	Basal (0 months) (N = 25)	After 6 months (N = 25)	p value
	Mean ± SD	Mean ± SD	​
HbA1C (mmol/L)	5.88 ± 0.89	5.59 ± 0.98	0.167
Cholesterol (mmol/L)	4.33 ± 0.67	4.43 ± 0.92	0.296
LDL (mg/dL)	85.85 ± 11.9	79.15 ± 13.5	**0.065**
HDL (mmol/L)	1.29 ± 0.24	1.09 ± 0.15	0.003*

**TABLE 16:** Effect of haloperidol on glycosylated hemoglobin and patients’ lipid profile after 6 months of treatment.

Blood glucose and lipid profile	Basal (0 months) (N = 25)	After 6 months (N = 25)	p value
	Mean ± SD	Mean ± SD	​
HbA1C (mmol/L)	5.86 ± 1.2	6.2 ± 2.1	0.583
Cholesterol (mmol/L)	4.2 ± 0.91	3.76 ± 0.85	0.131
LDL (mg/dL)	92.05 ± 22.9	79.4 ± 18.4	0.114
HDL (mmol/L)	1.28 ± 0.4	1.51 ± 1.74	0.50

Analyzed by Wilcoxon test.

*: Significant difference at P value <0.05.

**TABLE 17:** Effect of olanzapine + haloperidol on glycosylated hemoglobin and patients’ lipid profile after 6 months of treatment.

Blood glucose and lipid profile	Basal (0 months) (N = 25)	After 6 months (N = 25)	p value
	Mean ± SD	Mean ± SD	​
HbA1C (mmol/L)	5.90 ± 1.22	5.8 ± 1.72	0.937
Cholesterol (mmol/L)	4.41 ± 0.95	3.87 ± 0.87	0.594
LDL (mg/dL)	90.65 ± 17.9	85.4 ± 29.5	0.066
HDL (mmol/L)	1.48 ± 0.32	1.17 ± 0.29	0.686

Analyzed by Wilcoxon test.

*: Significant difference at P value <0.05.

### 3.7 Effect on renal and hepatic functions

As presented in Tables 18–22, no significant differences were observed among the study groups in baseline renal or hepatic function parameters. After 6 months of treatment with OLZ, HALP, or OLZ + HALP, renal assessment revealed a significant increase in both creatinine and urea concentrations in the OLZ + HALP group compared to the OLZ and HALP groups, whereas hepatic function parameters showed no significant differences among the treated groups.

**TABLE 18:** Renal and Hepatic function tests before starting Olanzapine, Haloperidol, and Olanzapine + Haloperidol treatment.

Renal andliver function Basal (0 months)	Olanzapine group (I) (N = 25)	Haloperidol group (II) (N = 25)	Olanzapine + haloperidol group (III) (N = 25)	
	Mean ± SD	Mean ± SD	Mean ± SD	
Creatinine (µmol/L)	77.38 ± 7.7	76 ± 8.67	76 ± 8.67	
Urea (mmol/L)	4.21 ± 0.71	4.18 ± 0.98	3.99 ± 0.98	
Total bilirubin (µmol/L)	7.84 ± 1.14	7.91 ± 1.70	7.74 ± 1.76	
Direct bilirubin (µmol/L)	2.46 ± 0.56	2.51 ± 0.73	2.50 ± 0.71	
ALT (U/L)	16.33 ± 3.05	16.26 ± 4.1	16.06 ± 4.5	
AST (U/L)	26.15 ± 6.5	21.35 ± 5.4	23.05 ± 4.4	
Alkaline phosphatase (U/L)	65.07 ± 11.4	61.88 ± 9.9	60.65 ± 8.9	
Albumin (g/L)	40.05 ± 2.11	42.21 ± 3.11	40.25 ± 3.56	
P value				
​	All groups	I vs. II	I vs. III	II vs. III
BUN	0.981	0.887	0.887	>0.99
Creatinine (µmol/L)	0.955	0.843	0.843	>0.99
Urea (mmol/L)	0.954	0.423	0.423	>0.99
Total bilirubin (µmol/L)	0.599	0.897	0.897	>0.99
Direct bilirubin (µmol/L)	0.497	0.346	0.346	>0.99
ALT (U/L)	0.825	0.162	0.162	>0.99
AST (U/L)	0.925	>0.99	>0.99	>0.99
Alkaline phosphatase (U/L)	0.196	0.252	0.252	>0.99

Analyzed by Mann-Whitney U test and Kruskal Wallis Test.

*: Significant difference at P value <0.05.

**TABLE 19:** Renal and hepatic function tests after 6 months of olanzapine, haloperidol, and olanzapine + Haloperidol treatment.

Renal andliver function (post 6 months)	Olanzapine group (I) (N = 25)	Haloperidol group (II) (N = 25)	Olanzapine + haloperidol group (III) (N = 25)	
	Mean ± SD	Mean ± SD	Mean ± SD	
Creatinine (µmol/L)	69.42 ± 12.7	68.26 ± 7.1	81.3 ± 9.3	
Urea (mmol/L)	4.65 ± 2	3.91 ± 1.18	7.4 ± 1.7	
Total bilirubin (µmol/L)	6.45 ± 2.09	6.18 ± 1.39	7.76 ± 1.69	
Direct bilirubin (µmol/L)	2.11 ± 1.09	2.3 ± 0.82	2.1 ± 0.95	
ALT (U/L)	13.35 ± 6.24	17.4 ± 6.1	14.67 ± 8.8	
AST (U/L)	28.97 ± 5.39	19.09 ± 6.88	21 ± 6.03	
Alkaline phosphatase (U/L)	78.83 ± 19.0	77.87 ± 13.7	70.66 ± 16	
Albumin (g/L)	40.93 ± 3.26	38.62 ± 4	39.46 ± 3.87	
P value				
​	All groups	I vs. II	I vs. III	II vs. III
Creatinine (µmol/L)	0.0375*	0.833	0.029*	0.047*
Urea (mmol/L)	0.03*	0.336	0.035*	0.013*
Total bilirubin (µmol/L)	0.954	0.411	0.899	0.447
Direct bilirubin (µmol/L)	0.954	0.864	0.448	0.331
ALT (U/L)	0.709	0.832	0.593	0.594
AST (U/L)	0.478	0.077	0.965	0.647
Alkaline phosphatase (U/L)	0.867	0.833	0.264	0.63
Albumin (g/L)	0.196	0.336	0.763	0.913

Analyzed by Mann-Whitney U test and Kruskal Wallis Test.

*: Significant difference at P value <0.05.

**TABLE 20:** Renal and Hepatic function tests after 6 months of Olanzapine treatment.

Kidney and liver function test	Basal (0 months) (N = 25)	After 6 months (N = 25)	p value
	Mean ± SD	Mean ± SD	​
Creatinine (µmol/L)	77.38 ± 7.7	69.42 ± 12.7	0.06
Urea (mmol/L)	4.21 ± 0.71	4.65 ± 2	0.35
Total bilirubin (µmol/L)	7.84 ± 1.14	6.45 ± 2.09	0.008*
Direct bilirubin (µmol/L)	2.46 ± 0.56	2.11 ± 1.09	0.159
ALT (U/L)	16.33 ± 3.05	13.35 ± 6.24	0.263
AST (U/L)	26.15 ± 6.5	28.97 ± 5.39	0.54
Alkaline phosphatase (U/L)	65.07 ± 11.4	78.83 ± 19.0	0.006*
Albumin (g/L)	40.05 ± 2.11	40.93 ± 3.26	0.58

Analyzed by Wilcoxon test.

*: Significant difference at P value <0.05.

**TABLE 21:** Renal and hepatic function tests after 6 months of haloperidol treatment.

Kidney and liver function test	Basal (0 months) (N = 25)	After 6 months (N = 25)	p value
	Mean ± SD	Mean ± SD	​
Creatinine (µmol/L)	76 ± 8.67	68.26 ± 17.1	0.083
Urea (mmol/L)	4.18 ± 0.98	3.91 ± 1.18	0.656
Total bilirubin (µmol/L)	7.91 ± 1.70	6.18 ± 2.39	0.024*
Direct bilirubin (µmol/L)	2.51 ± 0.73	2.3 ± 0.82	0.003*
ALT (U/L)	16.26 ± 4.1	17.4 ± 13.1	0.433
AST (U/L)	21.35 ± 5.4	19.09 ± 6.88	0.062
Alkaline phosphatase (U/L)	61.88 ± 9.9	77.87 ± 23.7	0.123
Albumin (g/L)	42.21 ± 3.11	38.62 ± 4	0.091

Analyzed by Wilcoxon test.

*: Significant difference at P value <0.05.

**TABLE 22:** Renal and Hepatic function tests after 6 months of Olanzapine + Haloperidol treatment.

Kidney and liver function test	Basal (0 months) (N = 25)	After 6 months (N = 25)	p value
	Mean ± SD	Mean ± SD	​
Creatinine (µmol/L)	76 ± 8.67	81.3 ± 9.3	0.906
Urea (mmol/L)	3.99 ± 0.98	7.4 ± 1.7	0.594
Total bilirubin (µmol/L)	7.74 ± 1.76	7.76 ± 1.69	0.173
Direct bilirubin (µmol/L)	2.50 ± 0.71	2.1 ± 0.95	0.172
ALT (U/L)	16.06 ± 4.5	14.67 ± 8.8	0.754
AST (U/L)	23.05 ± 4.4	21 ± 6.03	0.929
Alkaline phosphatase (U/L)	60.65 ± 8.9	70.66 ± 16	0.31
Albumin (g/L)	40.25 ± 3.56	39.46 ± 3.87	0.063

Analyzed by Wilcoxon test.

*: Significant difference at P value <0.05.

In the OLZ-treated group, a significant decrease in total bilirubin concentration was observed, accompanied by a notable increase in alkaline phosphatase (ALP) activity after 6 months of treatment. In contrast, the HALP-treated group demonstrated a significant reduction in both total and direct bilirubin levels following 6 months of drug administration. Meanwhile, the OLZ + HALP combination group did not exhibit any significant alterations in renal or hepatic function parameters throughout the treatment period.

### 3.8 Effect on the patient’s body weight and waist circumference

As shown in Tables 23–28, no significant differences were observed among the study groups in baseline body weight or waist circumference measurements. After 6 months of drug administration, the OLZ-treated group demonstrated a significant and progressive increase in body weight—from 70.38 kg at baseline to 76.77 kg after 3 months and 81.7 kg after 6 months.

**TABLE 23:** patients’ body weight and waist circumference before starting treatments.

Basal (0 months)		Olanzapine group (N = 25)	Haloperidol group (II) (N = 25)	Olanzapine + Haloperidol group (III) (N = 25)
Weight (kg)	Mean ± SD	70.38 ± 15.1	74.14 ± 25.4	67.8 ± 18.3
Waist circumference (cm)	Mean ± SD	91.29 ± 16.0	93.3 ± 11.6	89.47 ± 15.6
P value				
​	All groups	I vs. II	I vs. III	II vs. III
Weight	0.137	0.456	0.083	0.116
Waist circumference	0.367	0.357	0.695	0.159

Analyzed by Mann-Whitney U test, Kruskal Wallis test, and chi square test.

*: Significant difference at P value <0.05.

**TABLE 24:** Effect of olanzapine, haloperidol, and olanzapine + haloperidol on patients’ body weight and waist circumference after 3 months of treatment.

Post (3 months)		Olanzapine group (N = 25)<	Haloperidol group (II) (N = 25)	Olanzapine + haloperidol group (III) (N = 25)
Weight (kg)	Mean ± SD	76.77 ± 13	72.25 ± 14.2	67.97 ± 16.1
Waist circumference (cm)	Mean ± SD	95.2 ± 18.3	94.17 ± 10.3	94.1 ± 18.7
P value				
​	All groups	I vs. II	I vs. III	II vs. III
Weight	0.109	0.285	0.044*	0.140
Waist circumference	0.661	0.49	0.925	0.386

Analyzed by Mann-Whitney U test, Kruskal Wallis test and chi square test.

*: Significant difference at P value <0.05.

**TABLE 25:** Effect of Olanzapine, Haloperidol, and Olanzapine + Haloperidol on patients’ body weight and waist circumference after 6 months of treatment.

Post (6 months)		Olanzapine group (N = 25)	Haloperidol group (II) (N = 25)	Olanzapine + haloperidol group (III) (N = 25)
Weight (kg)	Mean ± SD	81.7 ± 23.6	73.01 ± 24.7	71.7 ± 26.1
Waist circumference (cm)	Mean ± SD	98.82 ± 18.8	96.65 ± 9.81	98.9 ± 12.4
P value				
​	All groups	I vs. II	I vs. III	II vs. III
Weight	0.381	0.262	0.211	0.946
Waist circumference	0.953	0.839	0.766	0.903

Analyzed by Mann-Whitney U test, Kruskal Wallis test, and chi square test.

*: Significant difference at P value <0.05.

**TABLE 26:** patients’ body weight and waist circumference during olanzapine treatment.

Determined parameters	Basal (0 months) (N = 25)	After 3 months (N = 25)	After 6 months (N = 25)	p value
Weight (kg) ➢Mean ± SD	70.38 ± 15.1	76.77 ± 23	81.7 ± 23.6	<0.001*
Waist circumference (cm) ➢Mean ± SD	91.29 ± 16.0	95.2 ± 18.3	98.82 ± 18.8	<0.001*

Analyzed by the Friedman test and Chi square test.

*: Significant difference at P value <0.05.

**TABLE 27:** patients’ body weight and waist circumference during haloperidol treatment.

Determined parameters	Basal (0 months) (N = 25)	After 3 months (N = 25	After 6 months (N = 25)	p value
Weight (kg) ➢Mean ± SD ➢Range	71.14 ± 25.4 51–169.5	72.25 ± 24.2 53–169.5	73.01 ± 24.7 53–169.5	0.861
Waist circumference (cm) ➢Mean ± SD ➢Range	93.3 ± 11.6 73–112	94.17 ± 10.3 73–112	96.65 ± 9.81 78–115	0.819
Appetite N (%) ➢1 ➢2	20 (100%) 0 (0%)	20 (100%) 0 (0%)	20 (100%) 0 (0%)	-

Analyzed by the Friedman test.

*: Significant difference at P value <0.05.

**TABLE 28:** patients’ body weight and waist circumference during olanzapine + haloperidol treatment.

Determined parameters	Basal (0 months) (N = 25)	After 3 months (N = 25)	After 6 months (N = 25)	p value
Weight (kg) ➢Mean ± SD	63.8 ± 18.3	67.97 ± 25.1	71.7 ± 26.1	<0.001*
Waiste circumference (cm) ➢Mean ± SD	89.47 ± 15.6	94.1 ± 18.7	98.9 ± 20.4	<0.001*

Analyzed by Friedman test.

*: Significant difference at P value <0.05.

In contrast, patients in the HALP-treated group did not exhibit any significant changes in either body weight or waist circumference throughout the 6-month period. Meanwhile, the OLZ + HALP combination group showed a significant increase in both parameters, with body weight rising from 63.8 kg to 71.7 kg and waist circumference increasing from 89.47 cm to 98.9 cm after 6 months of treatment.

## 4 Discussion

Schizophrenia is a chronic psychiatric disorder that imposes profound physical, social, and economic burdens, yet its impact on public health remains insufficiently recognized (Tandon et al., 2024). The illness severely reduces patient productivity and entails substantial, ongoing costs associated with hospitalization, therapy, and rehabilitation (Kadakia et al., 2022). Characterized by early adult onset, persistent progression, debilitating symptoms, functional decline, and social marginalization, schizophrenia stands among the most destructive and economically demanding mental disorders (Velligan and Rao, 2023).

Long-term management of schizophrenia necessitates continuous administration of antipsychotic medications. However, challenges such as suboptimal efficacy, poor adherence, extrapyramidal symptoms, weight gain, and sedation often compromise consistent treatment compliance (Neumeier et al., 2021; Correll and Howes, 2021). Although atypical antipsychotics exhibit a lower incidence of extrapyramidal effects such as Parkinsonism, dystonia, akathisia, and tardive dyskinesia, they are associated with a higher risk of metabolic adverse effects (Kameg and Champion, 2022; Fonseca et al., 2023). Furthermore, the full therapeutic potential and systemic side effects of these medications remain insufficiently elucidated, underscoring the need for continued investigation into their metabolic and inflammatory impacts (Siafis et al., 2023; Speyer et al., 2025; Pillinger et al., 2023). The present study was therefore designed to investigate these effects in patients with schizophrenia, aiming to provide clinicians with evidence-based insights to better balance therapeutic efficacy and safety, adjust dosing strategies, and implement appropriate patient monitoring to optimize treatment outcomes.

Our 6-month prospective analysis revealed distinct, statistically significant systemic alterations across treatment groups. Metabolic and anthropometric monitoring showed that olanzapine (OLZ) and the olanzapine-haloperidol (OLZ + HALP) combination significantly increased body weight and waist circumference, whereas haloperidol (HALP) monotherapy resulted in no significant weight change but uniquely and significantly normalized dopamine and ghrelin levels while reducing elevated leptin concentrations. Cardiovascular and lipid assessments demonstrated that OLZ significantly prolonged the QTc interval, elevated systolic blood pressure, and reduced both LDL and HDL levels; conversely, HALP significantly increased CK-MB levels without altering blood pressure, and the combination regimen significantly prolonged QTc and reduced LDL. Regarding organ function and electrolytes, the combined regimen significantly elevated creatinine and urea levels compared to monotherapies and reduced serum calcium, sodium, and chloride; meanwhile, OLZ significantly increased alkaline phosphatase activity and reduced sodium and chloride, and HALP significantly lowered total and direct bilirubin as well as potassium and chloride. Furthermore, all treatment arms significantly reduced elevated serotonin levels and, in the exploratory cytokine analysis, significantly downregulated the pro-inflammatory mediators IL-6, IL-17, and TNF-α from baseline.

Our observation that olanzapine (alone or in combination) led to significant weight gain and increased waist circumference, whereas haloperidol did not, aligns strongly with established consensus. Multiple meta-analyses and prospective studies confirm that second-generation antipsychotics like olanzapine carry a high risk of metabolic syndrome and weight gain, while first-generation agents like haloperidol are generally weight-neutral or associated with minimal changes. Similarly, our finding of dyslipidemia (reduced HDL and LDL changes) in the olanzapine arm is consistent with its well-documented metabolic liability.

The reduction of pro-inflammatory cytokines (IL-6, TNF-α, IL-17) observed in our study supports the growing body of evidence that antipsychotics exert immunomodulatory effects. Recent meta-analyses have consistently reported that successful antipsychotic treatment is associated with decreased serum levels of IL-6 and TNF-α, suggesting that dampening inflammation may be a marker of therapeutic response. Regarding appetite-regulating hormones, our finding that olanzapine reduced ghrelin levels is consistent with physiological feedback mechanisms attempting to counteract drug-induced weight gain. However, our observation that haloperidol significantly reduced leptin levels contrasts with some literature, which often reports minimal or non-significant leptin changes with typical antipsychotics, suggesting this may be a specific feature of our cohort or related to the marked lack of weight gain in this group.

Our cardiovascular results present a nuanced comparison with the literature. While we observed significant QTc prolongation with olanzapine, some previous studies have suggested that haloperidol (particularly intravenous) poses a higher risk for QTc prolongation than oral olanzapine. The elevation in CK-MB levels seen in our haloperidol group, while not reaching the threshold for neuroleptic malignant syndrome, reflects known risks of transient enzyme elevations associated with typical antipsychotics. Finally, the significant increase in renal markers (creatinine, urea) observed in our combination group aligns with emerging evidence that polypharmacy in psychiatric patients can impose a greater cumulative burden on renal function than monotherapy.

The dopamine hypothesis remains central to understanding schizophrenia, proposing that dysregulation of dopamine transmission—particularly within the mesolimbic and prefrontal pathways—contributes to symptom development (Weinberger, 2022). However, subsequent research has identified additional roles for glutamate, GABA, acetylcholine, and serotonin in the disorder’s neurobiology (Rezaei, 2022; Bansal and Chatterjee, 2021). Although hyperdopaminergic signaling has long been linked to positive symptoms such as hallucinations and delusions—through excessive D2 receptor activation and cortical circuit disturbances (Kambeitz et al., 2014)—the relationship between dopamine dysregulation and the full clinical spectrum of schizophrenia remains incompletely understood (Brisch et al., 2014; Collo et al., 2020). The influence of antipsychotic medications on serum dopamine levels has not been consistently documented, with available evidence yielding mixed results. In the present study, patients with schizophrenia demonstrated slightly but non-significantly lower dopamine levels compared with healthy controls at baseline. Following 6 months of treatment, olanzapine administration resulted in an elevation of dopamine concentrations, whereas haloperidol treatment restored dopamine levels to near-normal values. Interestingly, patients receiving the olanzapine + haloperidol combination exhibited significantly lower dopamine levels than those treated with haloperidol alone, suggesting a possible pharmacodynamic interaction influencing dopaminergic modulation.

Serotonin (5-hydroxytryptamine, 5-HT) is integral to the regulation of mood, cognition, and psychotic symptoms associated with schizophrenia (Jiménez-Trejo et al., 2024). Consequently, assessing serum or whole-blood serotonin levels can offer valuable insights into serotonergic abnormalities underlying symptom severity, particularly with respect to negative and cognitive domains (Rojas Cabrera et al., 2023). Whole-blood serotonin concentrations may also serve as practical peripheral indicators of central serotonergic activity, facilitating non-invasive evaluation of neurochemical changes (Rojas Cabrera et al., 2023).

Previous research has demonstrated altered serotonin levels in patients with schizophrenia, often correlating with disease duration and symptom intensity, thereby suggesting a potential role for serotonin as a biomarker for disease progression and treatment response (Quednow et al., 2020). Although the clinical utility of serotonin measurement is still limited by methodological and interpretative challenges, it remains a promising avenue for improving diagnostic precision and therapeutic monitoring.

In the present study, schizophrenic patients exhibited significantly lower serotonin levels compared with healthy controls. Treatment with OLZ, HALP, and their combination (OLZ + HALP) for 6 months resulted in a significant restoration of serotonin concentrations to near-normal levels, highlighting the potential modulatory effect of these antipsychotic regimens on serotonergic function.

About 33% of persons diagnosed with schizophrenia also meet criteria for metabolic syndrome, with prevalence rates reaching up to 69% among those with chronic illness (Molina et al., 2021). The prevalence of obesity, type 2 diabetes, and hypercholesterolemia among patients diagnosed with schizophrenia is approximately 3–5 times higher than that of the general population (Dong et al., 2024). While numerous studies have investigated weight gain and metabolic alterations associated with various antipsychotic medications, the extent of these changes particularly for haloperidol (HALP) and Olanzepine (OLZ)- remains uncertain and inconsistent (Fabrazzo et al., 2015; Jin et al., 2024; Horska et al., 2024). Furthermore, the metabolic effects of the combined administration of both drugs including impacts on patient body weight and blood glucose levels have not been comprehensively studied, highlighting the need for further investigation.

Studies examining the effects of OLZ and HALP on blood glucose levels have produced conflicting results, largely due to their distinct pharmacological mechanisms. Several studies report that OLZ administration is associated with hyperglycemia, new-onset diabetes, and in rare cases diabetic ketoacidosis (Toledo et al., 2022). Olanzapine may impair insulin-mediated glucose disposal and increase insulin resistance without necessarily causing immediate weight gain (Toledo et al., 2022). Mechanistically, OLZ can reduce pancreatic beta cells responsiveness via 5-HT1 antagonism and inhibit insulin signaling pathways, contributing to glucose dysregulation. Some studies suggest biphasic changes in insulin secretion, where olanzapine initially alter insulin release and subsequently induces insulin resistance (Meyer, 2002; Klingerman et al., 2015; Chintoh et al., 2008).

On the other hand, HALP appears to exert less pronounced or inconsistent effects on blood glucose. Short-term administration often shows no significant impact on glucose levels compared to placebo (van Keulen et al., 2018). Although isolated reports indicate that HALP may impair blood glucose control by reducing insulin efficacy or, in rare cases causing hypoglycemia (Vidarsdottir et al., 2010). In the present study, no significant changes in the HbA1C concentration were observed following 6 months of OLZ, and OLZ + HALP administration. Conversely the HALP-treated group exhibited a noticeable increase in HbA1C levels.

Ghrelin and leptin are among the most significant hormones involved in regulating metabolic processes and fat deposition in the human body (Cui et al., 2017). Traditionally, Leptin is recognized for its central role in regulation of hunger, neuroendocrine activity, and energy homeostasis; however, accumulating evidence suggests that it also participates in broader physiological processes (Vijayashankar et al., 2024). Including metabolic processes, endocrine regulation, and immunological functions (Ghomraoui et al., 2017; Al-Hussaniy et al., 2021). Ghrelin is a peptide hormone recognized for its function in appetite regulation, fat storage, inhibition of insulin secretion, stimulation of growth hormone release, feeding behavior, and energy homeostasis (Abdalla, 2015). Both Leptin and ghrelin exhibit pleiotropic effects on the central nervous system; for instance, evidence from clinical studies on patients with depression indicates that these hormones may exert antidepressant-like effects, as their circulating levels often differ between depressed patients and healthy controls (Zarouna et al., 2015). Nonetheless, findings remain inconsistent–some studies report positive correlations between elevated hormone levels and mood or anxiety disorders, whereas others demonstrate that depressive patients show reduced ghrelin and leptin levels both before and after treatment compared with controls (Kishi and Elmquist, 2005).

Despite the clear clinical relevance, few studies have examined the impact of antipsychotic medications on ghrelin and leptin concentrations. Antipsychotics are well known for inducing metabolic side effects, such as weight gain and altered glucose and lipid metabolism, which may stem from dysregulation of these hormones. The limited available evidence underscores the necessity for further research to elucidate how different antipsychotic agents modulate ghrelin and leptin activity and contribute to metabolic disturbances in patients with schizophrenia.

In the current study, we observed significantly lower ghrelin levels in patients with schizophrenia compared with healthy individuals. Treatment with OLZ did not significantly alter ghrelin concentrations; however, HALP administration for 6 months led to a notable increase in serum ghrelin levels. Moreover, combined OLZ + HALP treatment resulted in higher ghrelin levels than OLZ monotherapy, though still lower than those observed in the HALP-treated group. Regarding leptin, schizophrenic patients demonstrated markedly elevated baseline levels compared with healthy controls. These concentrations were largely unaffected by OLZ or the combined regimen, whereas HALP treatment significantly reduced serum leptin levels, indicating a distinct modulatory effect on this hormone.

The assessment of leptin concentrations can provide valuable insights into the regulation of energy balance. Variability in the reported findings may be attributed to differences in study populations (e.g., healthy volunteers versus patients), treatment duration (acute versus chronic), dosage regimens, and baseline metabolic status of participants. Such inconsistencies indicate that involvement of distinct underlying mechanisms and underscore the necessity for further research to elucidate the metabolic effects of OLZ, HALP, and their combinations.

Antipsychotics are generally associated with a tendency to induce dyslipidemia, characterized by elevations in triglycerides (TG), total cholesterol (TC), and low-density lipoprotein cholesterol (LDL-C) (Li et al., 2020). The underlying mechanisms for Olanzapine may involve enhanced phosphorylation promoting lipogenesis, and insulin resistance impairing lipoprotein lipase activity, and antagonism of hypothalamic 5-HT and histamine H1 receptors, collectively leading to increased appetite and weight gain, enhanced AMPK activity, causing slower LDL catabolism and increased TG production (Chen et al., 2018; Siafis et al., 2018; Singh et al., 2020). However, Haloperidol’s effects on blood lipids are less consistent and less pronounced than olanzapine’s. Some studies suggest that haloperidol inhibits cholesterol biosynthesis by interfering with enzymes involved in sterol metabolism, resulting in the accumulation of sterol intermediates and reduced cholesterol formation (Chaggar et al., 2011; Vidarsdottir et al., 2010). In the present study, OLZ administration did not significantly alter cholesterol or LDL levels although a significant reduction in the HDL was observed compared baseline Conversely, haloperidol treatment led to a notable reduction in cholesterol and LDL levels accompanied by an increase in the HDL level. The combined administration of OLZ + HALP resulted in higher cholesterol and LDL levels compared to HALP monotherapy.

Olanzapine is widely recognized to cause significant weight gain in patients treated for schizophrenia or other psychiatric disorders. Mechanistically, this effect is primarily mediated through increased appetite and hyperphagia resulting from antagonism of hypothalamic receptors (5-HT2C, histamine H1) and secondary metabolic effects (Roerig et al., 2011; Huang et al., 2020; Samy et al., 2021). The impact of Haloperidol on body weight is less consistent. Some studies, particularly in animal models, report no significant weight gain or even weight loss (Mourra et al., 2020; Vidarsdottir et al., 2010). Other clinical data indicate moderate weight gain with haloperidol, but generally less pronounced than those observed with olanzepine or other atypical antipsychotics (McGrane et al., 2022; Ibragimov et al., 2024). In the present study, patients with schizophrenia who received OLZ or OLZ + HALP for 6 months exhibited a significant increase in their body weight and waist circumference following which was not evident after 3 months of drugs treatment. On the other hand, treatment with HALP for 6 months did not result in a significant changes body weight or waist circumference, highlighting the differential metabolic impact of these antipsychotic regimens.

Olanzapine has occasionally been associated with electrolyte disturbances most notably hyponatremia, which is often linked to the syndrome of inappropriate antidiuretic hormone secretion (SIADH) (Sachdeva and Choudhary, 2017; Antidiüretik, 2016). Pharmacodynamic interaction data indicate that olanzapine may exhibit synergistic effects with calcium, magnesium, potassium, and sodium oxybates, potentially altering their serum effects (Mohamed et al., 2017; Manolis et al., 2019). However, direct and conclusive evidence demonstrating consistent alterations in baseline blood electrolyte levels induced by olanzapine remains limited.

In contrast, Haloperidol treatment has been reported to cause a significant decrease in plasma magnesium concentrations, although changes in calcium or phosphorus levels have not been consistently observed (Jabotinsky-Rubin et al., 1993; Kronbauer et al., 2019). The effects of haloperidol on sodium, potassium, calcium, and chloride are less well documented and studies in this field are insufficient. In the present study, 6 months administration of OLZ resulted in a significant reduction in both sodium and chloride ions, and magnesium concentration remained largely unaffected. Conversely, HALP treatment produced a significant decline in chloride ion without markedly affecting other electrolytes. Notably, the combined administration of OLZ + HALP led to significant reduction in sodium, calcium, and chloride levels, whereas potassium and magnesium serum concentrations did not show substantial alterations.

Both Olanzapine and haloperidol exert measurable effects on renal and hepatic function, although they differ in nature, extent, and clinical significance of these alterations. Olanzapine undergoes extensive hepatic metabolism, with less than 10% of the administered dose excreted unchanged by the kidneys (Mao et al., 2023), Renal impairment has been shown to slightly prolong olanzapine’s half-life and increase systemic exposure (Sun et al., 2019). Despite its limited renal elimination, olanzapine has been occasionally associated with acute kidney injury (AKI), potentially secondary to neuroleptic malignant syndrome, hypotension, or urinary retention during therapy. Nevertheless, direct nephrotoxic effects are uncommon (Bilgic et al., 2018; Ong et al., 2024).

Mild, transient, and asymptomatic elevations in liver aminotransferases (ALT, AST) have been reported in some patients treated with OLZ; however, clinically significant hepatotoxicity remains rare (Slim et al., 2016). The hepatic risk may be greater in individuals with preexisting liver diseases such as viral hepatitis, where olanzapine could exacerbate liver injury (Telles-correia et al., 2017). Experimental studies in animal models have further demonstrated that OLZ can induce hepatocellular injury, inflammation, and lipid accumulation, suggesting a potential for hepatic steatosis and hepatotoxicity (Shah et al., 2016). In the present study, 6 months of OLZ administration did not result in significant changes in renal function parameters (Urea and creatinine determination). However, regarding hepatic indices, OLZ produced a significant reduction in total bilirubin blood concentration accompanied by a marked elevation in alkaline phosphatase activity.

Haloperidol, on the other hand, has shown dose-dependent renal toxicity in animal studies, including tubular damage, vascular dilation, increased glomerular size, and histological abnormalities after chronic high-dose treatment (Akintunde et al., 2017). Although Clinical evidence of haloperidol-induced nephrotoxicity is limited, some reports suggests potential renal adverse effects with prolonged or high-dose use (Damba et al., 2022). Haloperidol has also been associated with asymptomatic elevations in liver enzymes (ALT, AST, ALP, bilirubin) among patients with schizophrenia, though clinically significant hepatotoxicity is uncommon (Gunther and Dopheide, 2023; Mulia, 2022). In our findings, 6 months of HALP administration did not produce a significant alteration in renal function tests but was associated with a noticeable reduction in both total and direct bilirubin concentrations. Conversely, combined administration of OLZ + HALP did not result in any significant changes in both renal and hepatic functions.

Both Olanzapine and haloperidol exert notable effects on the cardiovascular system; however, these effects differ in their nature, underlying mechanisms, and clinical outcomes. Despite some overlapping risks and contradictory findings, available research on their cardiovascular impact remains limited. OLZ has been strongly associated with significant cardiovascular risks largely linked to its metabolic side effects, including weight gain, insulin resistance, and consequent increased risk of hypertension and atherosclerosis (Chen et al., 2018; Jin et al., 2024). Several Studies have also suggested that olanzapine may directly induce cardiomyopathy. The proposed mechanisms involve metabolic stress and receptor antagonism affecting cardiac cells (Tolu-Akinnawo et al., 2024; Gulac et al., 2020). Furthermore, acute administration in animal models has demonstrated that olanzapine can lower blood pressure, decrease venous tone, and reduce cardiac contractile function, possibly through interactions with muscarinic, α-adrenergic, and histamine H1 receptors (Gulac et al., 2020). Collectively, OLZ poses a substantial cardiovascular risk, warranting more comprehensive and systematic investigations.

While haloperidol is a known QT-prolonging agent, significant prolongation and torsades de pointes are most frequently associated with intravenous administration or high doses. Our findings of a modest ∼12 m increase with 10 mg/day oral haloperidol align with previous studies showing that oral formulations carry a lower risk than IV haloperidol (Schrijver et al., 2018; Miceli et al., 2010; Beach et al., 2018). Hemodynamically, haloperidol has been shown to causes vasodilation, leading to reduced systemic vascular resistance and blood pressure. It may also reduce heart rate and alter heart rate variability, occasionally resulting in bradycardia (Vandael et al., 2016). In the current study, ECG (QTc) assessment revealed a significant prolongation in QTc interval in both OLZ and combined OLZ + HALP treatment groups, which may pose potential cardiac risks in vulnerable patients. In contrast, HALP alone did not significantly affect QTc interval. Additionally, OLZ administration was associated with a significant increase in SBP, a change not observed in HALP or OLZ + HALP treated groups.

## 5 Conclusion

This study provides a comprehensive evaluation of the systemic, metabolic, and inflammatory consequences of haloperidol, olanzapine, and their combined administration in schizophrenic patients, extending beyond conventional assessments of therapeutic efficacy. Haloperidol monotherapy demonstrated higher metabolic safety, with no significant weight gain or lipid disturbances over 6 months, and uniquely normalized dopamine levels and appetite-regulating hormones (increased ghrelin, decreased leptin), suggesting favorable neurochemical restoration in patients with predominant negative symptoms. However, this regimen was associated with modest CK-MB elevation and electrolyte shifts requiring monitoring. In contrast, olanzapine monotherapy, while effectively reducing serotonin and providing broad receptor antagonism, imposed metabolic and cardiovascular costs: substantial weight gain, increased waist circumference, dyslipidemia, QTc prolongation, and elevated systolic blood pressure.

These findings position haloperidol as the preferred option for patients at high metabolic risk or with pre-existing cardiovascular disease, whereas olanzapine may be reserved for those requiring broader symptom control who can tolerate intensive metabolic monitoring. The olanzapine-haloperidol combination presented a complex risk-benefit calculus. On the benefit side, it elicited the most robust anti-inflammatory response, showing the greatest reductions in IL-6, IL-17, and TNF-α signals in our exploratory subsample, and produced more favorable lipid changes than olanzapine alone (modest LDL reduction without the pronounced HDL decrease). However, combination therapy imposed unique safety concerns: elevated creatinine and urea levels (indicating cumulative renal burden not seen with either monotherapy), sustained QTc prolongation, and pronounced electrolyte disturbances alongside weight gain comparable to olanzapine monotherapy.

These findings suggest that while combination therapy may offer enhanced anti-inflammatory and potentially neuroprotective effects particularly valuable in treatment-resistant or high-inflammatory phenotypes, it requires stringent renal function surveillance and cardiac monitoring. These findings support a precision psychiatry approach wherein treatment selection is guided not solely by symptom profiles but by integrated assessment of metabolic, cardiovascular, renal, and inflammatory status. Future studies should validate these biomarkers prospectively and explore whether early intervention based on these systemic signatures can prevent treatment-emergent complications and improve long-term outcomes in schizophrenia management.

### 5.1 Limitation

The present study has certain limitations. First, the treatment allocation was naturalistic and based on clinical judgment rather than randomization. Consequently, patients in the combination therapy group may have had higher illness severity (treatment resistance) compared to monotherapy groups. While baseline metabolic and physiological parameters were comparable across all groups, the potential for confounding by indication regarding psychiatric severity cannot be fully excluded. Cytokine results represent semi-quantitative estimates of relative changes rather than absolute concentrations, and that validation with ELISA would strengthen future studies. Our exploratory biochemical analyses revealed several potentially important patterns (electrolyte alterations, lipid changes, neurotransmitter shifts), but given the large number of comparisons without multiplicity correction, these findings should be considered hypothesis-generating and require independent replication before clinical interpretation. Future studies should prospectively validate these exploratory biomarker findings in independent cohorts with appropriate sample size calculations and pre-registration of hypotheses. Because cytokines were assessed by Western blot densitometry in a small subset (n = 12), these results should be considered semi-quantitative and hypothesis-generating, and require confirmation using larger samples and quantitative cytokine assays.

## Data Availability

The original contributions presented in the study are included in the article/supplementary material, further inquiries can be directed to the corresponding author.
